# A New Mixed-Gas-Detection Method Based on a Support Vector Machine Optimized by a Sparrow Search Algorithm

**DOI:** 10.3390/s22228977

**Published:** 2022-11-20

**Authors:** Haitao Zhang, Yaozhen Han

**Affiliations:** School of Information Science and Electrical Engineering, Shandong Jiaotong University, Jinan 250357, China

**Keywords:** mixed-gas detection, support vector machine, sparrow search algorithm, prediction, classification

## Abstract

To solve the problem of the low recognition rate of mixed gases and consider the phenomenon of low prediction accuracy when traditional gas-concentration-prediction methods deal with nonlinear data, this paper proposes a mixed-gas identification and gas-concentration-prediction method based on a support vector machine (SVM) optimized by a sparrow search algorithm (SSA). Principal component analysis (PCA) is applied to perform data dimensionality reduction on the input data, and SSA is adopted to optimize the SVM hyperparameters to improve the recognition rate and gas-concentration-prediction accuracy of mixed gases. For the mixed-gas identification, the classification accuracy is significantly improved under the proposed SSA optimization SVM method (SSA-SVM), compared with random forest (RF), extreme-learning machine (ELM), and BP neural network methods. With respect to gas-concentration prediction, the maximum fitting degrees reached 99.34% for single gas-concentration prediction and 97.55% for mixed-gas-concentration prediction. The experimental results show that the SSA-SVM method had a high recognition rate and high concentration-prediction accuracy in gas-mixture detection.

## 1. Introduction

Mixed-gas detection is of great significance in the fields of food safety testing [1], agricultural production management [2], urban environmental quality testing [3], and life science research [4], particularly in industrial production. This is one of the key means to prevent fire, explosion, poisoning, and other safety accidents and ensure the safe production of enterprises. For example, once flammable and explosive gases, such as methane and ethylene, leak, it is likely to cause fire, explosion, and other safety accidents.

Gas sensors are an important component of gas detection. Some scholars focus on improving the performance of gas sensors to enhance the detection level of mixed gases. For instance, metals were doped into nanomaterials to raise the sensitivity of sensors in [5,6]. Polyaniline/single-walled carbon nanotube composites were utilized to increase the selectivity and stability of the sensor at room temperature in [7]. The soft-film-plate method and nano-casting strategy were adopted to enhance the sensitivity, response, and recovery time of porous metal oxide sensors in chemical synthesis in [8].

The performance of gas sensors has been promoted through composites, preparation processes, and doping. However, it is far from enough to consider only the hardware. As a multi-disciplinary interdisciplinary subject, machine learning is a research hotspot in the field of artificial intelligence and pattern recognition. Its application covers all fields of artificial intelligence [9,10,11]. Machine learning is also applied in the field of mixed-gas detection. Peng et al. [12] proposed a mixed-gas-identification method based on a deep convolutional neural network (DCNN), as DCNNs have high accuracy and robustness.

Due to the complex network structure of DCNNs and the possibility of falling into local optima, Zhang et al. [13] adopted a BP neural network with a simple network structure to realize the anti-interference detection of carbon monoxide and methane. However, the optimization objective function of the network was complex, which led to a slow convergence speed, and it was difficult to achieve timeliness in practical applications.

For sake of further improving the time efficiency of gas detection, random forest (RF), and k-nearest neighbor (KNN) algorithms were adopted [14,15]. Researchers experimentally demonstrated that the response time of gas detection is reduced to an extent under RF and KNN methods. Yet, with the increase in training samples, the amount of calculation for the KNN becomes larger, and its fault tolerance to training data is poor. The RF is overly sensitive to small changes in the training dataset and tends to overfit during classification [16].

Support vector machines (SVMs), as a type of machine-learning algorithm, has simple calculations, strong versatility, and robustness. SVMs have been widely applied in pattern classification and nonlinear regression, such as image recognition [17,18], text classification [19], fault detection [20,21], etc. In the field of mixed-gas detection, Rachid et al. [22] compared the effectiveness of partial least squares with the SVM method in monitoring gas concentrations in a confined environment. The results showed that gas concentrations were more accurately estimated under the SVM method. Zhao et al. [23] adopted a BP neural network, SVM, and extreme-learning machine (ELM) for identifying ethanol, acetone, and formaldehyde.

The results showed that SVM had the highest recognition accuracy. Zhang et al. [24] achieved the semi-quantitative detection of toxic and harmful gas mixtures in the kitchen through electronic nose detection. Whether through pattern classification or nonlinear regression, the relevant parameters of the SVM (mainly penalty parameter *c* and kernel function parameter *g*) must be adjusted to obtain a relatively good prediction effect. The grid search method is commonly used to optimize the selection of SVM parameters [25].

Although the global optimal solution can be found by grid search, the hyperparameter space is not limited. If the scope is expanded to find the global optimal solution, it will be time-consuming. Scholars have proposed different optimization algorithms to solve the problem of SVM hyperparameters, including genetic algorithms (GA) [26,27], particle swarm optimization (PSO) algorithms [28,29], ant colony algorithms [30], grey wolf optimization (GWO) algorithms [31], etc.

In the field of mixed-gas detection, the hyperparameters of support vector regression (SVR) are optimized by PSO to improve the prediction accuracy of mixed gas [32]. Deng et al. [33] proposed a GWO optimization SVM method to effectively suppress the effects of temperature and pressure on carbon monoxide. Li et al. [34] presented the SVR method optimized by GA to increase the prediction accuracy of the gas content in the drilling site. This method achieved precise control of the differences outside the prediction point.

The above optimization algorithms have enhanced the performance of SVM/SVR to an extent. However, PSO and GWO tend to fall into local optima when dealing with complex problems, and the convergence speed is slow in late iterations. The efficiency of GA is usually lower when compared with other traditional optimization algorithms.

Sparrow search algorithm (SSA), a new swarm intelligence optimization algorithm, was proposed by Xue and Shen in 2020 [35]. The algorithm mainly simulates the foraging and anti-predation behavior of sparrows. SSA has attracted wide attention because of its fast convergence speed and strong optimization ability. Wang et al. [36] adopted SSA to solve the optimal configuration model of distributed generations, and the effectiveness and superiority of SSA were verified by experimental simulation.

Song et al. [37] described the SSA optimization ELM method for the evaluation of water quality, which successfully overcomes the instability and nonlinearity of water quality parameter data. Liu et al. [38] optimized the SSA method to solve the problem of unmanned aerial vehicle (UVA) path planning. The modified SSA provides the best route for UAVs in complex 3D flight environments.

In the field of image processing, Wu et al. [39] proposed a modified SSA algorithm to deal with the matter of poor performance of threshold image segmentation in peak-to-noise ratio and feature similarity. Zhang et al. [40] introduced SSA into an adaptive enhancement classifier to improve the potential of lung CT image classification performance and the probability of early-stage cancer detection. Aiming at the problem of a low recognition rate of mixed gas and low prediction accuracy of gas concentration, this paper proposes a mixed-gas-detection method based on SSA-SVM. Its contribution is summarized as follows.

(1) An optimization algorithm based on the sparrow search algorithm to optimize the hyperparameters of support vector machines is designed for the identification of mixed gases and the prediction of gas concentration.

(2) The parameter selection of SVM/SVR is solved by the SSA algorithm. In mixed-gas identification, the identification accuracy of the mixed gas is improved by combining PCA with SSA-SVM. In gas-concentration prediction, the fitting degree of gas-concentration prediction under single gas and mixed gas is improved by the SSA-SVR. Compared with GWO and GA optimization algorithms, the SSA optimization algorithm improves the convergence speed and prediction fitting degree.

The structure of this paper is organized as follows. Section 2 introduces the mixed-gas-detection method. In Section 3, the mixed-gas identification and gas-concentration prediction are experimentally verified, and the results are analyzed and compared. Finally, our summary is given in Section 4.

## 2. Mixed-Gas-Detection Methods

**Figure 1:**
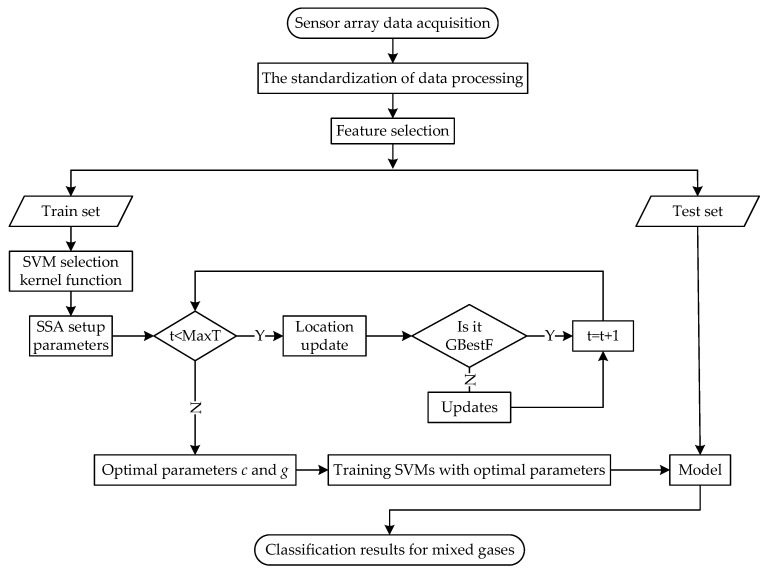
The SSA-SVM mixed-gas-identification method.

**Figure 2:**
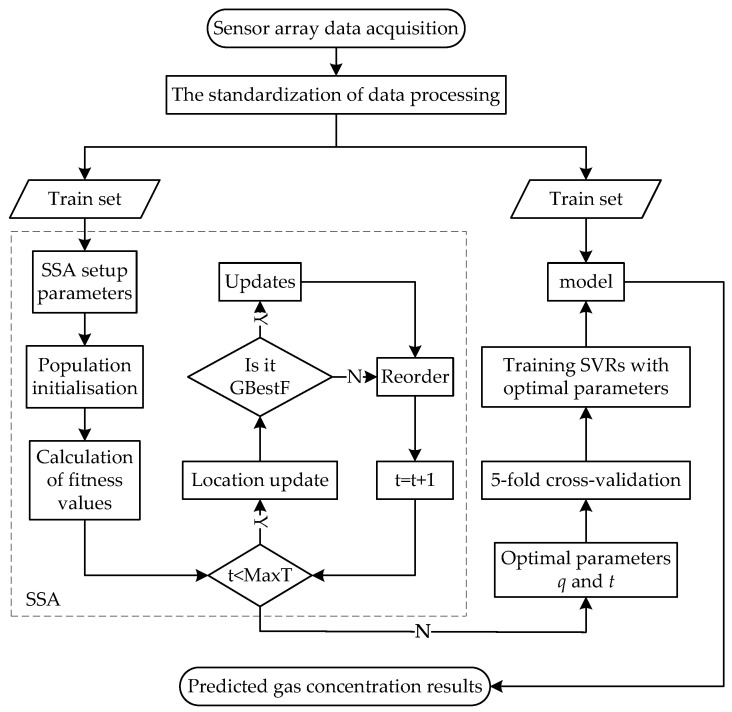
The SSA-SVR mixed-gas-concentration-prediction method.

In order to further improve the accuracy of mixed-gas identification and concentration prediction, a new mixed-gas-detection method based on SSA-SVM is proposed in this paper. The flow chart of mixed-gas identification based on SSA-SVM is shown in Figure 1, and the flow chart of mixed-gas-concentration prediction based on SSA-SVR is shown in Figure 2. The dataset provided by the University of California Irvine (UCI) machine-learning repository is adopted for simulation tests in MATLAB 2021 b. This section presents the proposed method in detail.

### 2.1. Mixed-Gas Classification and Gas-Concentration Prediction Based on SVM

#### 2.1.1. Mixed-Gas Classification

SVM, as a machine-learning algorithm, is commonly used to realize the pattern recognition of gas sensor arrays. The main idea is to construct a classification hyperplane as a decision surface to distinguish mixed gases. The relationship between a gas sensor and gas mixture is usually nonlinear, such that a correctly divided hyperplane is often not found in the original gas sample space. Thus, the original low-dimensional space is mapped into a high-dimensional space to search for a suitable partition hyperplane.

In the experiment, the mixed-gas classification dataset has 41,785 samples. Then, the training set in the given feature space is represented as

(1) $$D=\left\{\left(x_{1},y_{1}\right),\ldots ,\left(x_{41785},y_{41785}\right)\right\}\in {\left(X\times Y\right)}^{41785}$$

where $x_{i}\in X,y_{i}\in Y=\left\{1,2,3,4\right\}\left(i=1,2,\ldots ,41785\right)$; $x_{i}$ is the main feature vector extracted by PCA; $y_{i}$ is the gas classification label.

The optimal hyperplane of high-dimensional space can be defined as

(2) $$f\left(x\right)=w^{*}\varphi \left(x\right)+b$$

where *w* is the normal vector of the classification hyperplane and *b* is the intercept.

After the input data is mapped to a high-dimensional space, the SVM optimization problem becomes

(3) $$\min_{w,b}\frac{1}{2}{∥w∥}^{2}+c\sum_{i=1}^{41785}\xi_{i}$$

where *c* is the regularization coefficient and $\xi_{i}$ is the relaxation variable.

SVM maps linear and indivisible low-dimensional space to high-dimensional space by nonlinear transformation, and it has “dimension disaster” when operating in high-dimensional space. The introduction of the kernel function can not only transform the feature from low-dimensional space to high-dimensional space but also effectively avoid “dimension disaster” and reduce the amount of computation. For optimization problems, it is necessary to select an appropriate kernel function $K(x,x_{i})$ and penalty parameter *c* and then to construct and solve the optimization problem.

(4) $$\begin{array}{c} \max Q\left(\lambda \right)=\sum_{i=1}^{m}\lambda_{i}-\frac{1}{2}\sum_{i=1}^{m}\sum_{j=1}^{m}\lambda_{i}\lambda_{j}y_{i}y_{j}K\left(x_{i},x_{j}\right)\\ s.t\left\{\begin{array}{c} \sum_{i=1}^{m}\lambda_{i}y_{i}=0\\ 0\leq \lambda_{i}\leq c \end{array}\right.,i=1,2,\ldots ,m \end{array}$$

Then, the optimal solution is obtained as $\lambda^{*}={\left({\lambda_{1}}^{*},{\lambda_{2}}^{*},\ldots ,{\lambda_{m}}^{*}\right)}^{T}$.

According to the optimal solution, $\lambda^{*}$, $w^{*}$ is calculated as

(5) $$w^{*}=\sum_{i=1}^{m}\lambda_{i}^{*}y_{i}K\left(x,x_{i}\right)$$

To choose a positive component $\lambda_{j}^{*}$ ($0<\lambda_{j}^{*}<C$) of $\lambda^{*}$, the threshold is calculated as

(6) $$b^{*}=y_{i}-\sum_{i=1}^{m}y_{i}\lambda_{i}^{*}K\left(x_{i}-x_{j}\right)$$

Finally, the decision function is constructed as

(7) $$f\left(x\right)=\mathrm{sgn}(\sum_{i=1}^{m}\lambda_{i}^{*}y_{i}K\left(x,x_{i}\right)+b^{*})$$

#### 2.1.2. Mixed-Gas-Concentration Prediction

SVR, as an important application branch of SVM, is superior to other machine-learning algorithms in gas-concentration prediction. The basic idea of SVR is no longer to find an optimal classification surface to separate the samples but to find an optimal classification surface to minimize the error of all training samples from the optimal classification surface.

For the regression problem, the training data $T=\left\{\left(x_{1},y_{1}\right),\;\ldots ,\left(x_{n},y_{n}\right)\right\},y_{i}\in R$ is given to make $f\left(x\right)=w^{T}x+b$ and $y_{i}$ as close as possible. Then, the loss is calculated from the difference between the predicted output gas concentration f(x) and the real gas concentration $y_{i}$. The corresponding loss function is

(8) $$L\left(z\right)=\max\left(0,\left|z\right|-\varepsilon \right)=\left\{\begin{array}{c} 0,\begin{array}{ccc} & & if\left|z\right|<\varepsilon \end{array}\\ \left|z\right|-\varepsilon ,\begin{array}{cc} & otherwise \end{array} \end{array}\right.$$

Thus, the optimization problem of SVR can be expressed as

(9) $$\begin{array}{c} \min_{w,b}\frac{1}{2}{∥w∥}^{2}+C\sum_{i=1}^{n}L(z)\\ s.t.\begin{array}{cc} & \left|y_{i}-f\left(x\right)\right| \end{array}\leq \varepsilon \end{array}$$

Slack variables $\xi_{i}$ and ${\xi_{i}}^{*}$ are then introduced to replace the loss function, and Equation (9) is rewritten as

(10) $$\begin{array}{c} \min_{w,b}\frac{1}{2}{\left\|w\right\|}^{2}+C\sum_{i=1}^{n}\left(\xi_{i}+\xi_{i}^{*}\right)\\ s.t.\left\{\begin{array}{c} y_{i}-f\left(x_{i}\right)\leq \varepsilon +\xi_{i}\\ f\left(x_{i}\right)-y_{i}\leq \varepsilon +\xi_{i}^{*}\\ \xi_{i},\xi_{i}^{*}\geq 0 \end{array}\right. \end{array}$$

where *C* is the regularization coefficient, which is mainly used to prevent SVR overfitting. If the value of is *C* too large or too small, the generalization ability of SVR will be deteriorated.

Then, the Lagrange multiplier is introduced to obtain the Lagrange function and the dual problem. The solution of SVR can be obtained by solving the dual problem.

(11) $$f\left(x\right)=\sum_{i=1}^{n}\left(\lambda_{i}^{*}-\lambda_{i}\right){x_{i}}^{T}x_{j}+b$$

Finally, the kernel function is added to obtain the decision function of the gas-concentration prediction.

(12) $$f\left(x\right)=\sum_{i=1}^{n}\left(\lambda_{i}^{*}-\lambda_{i}\right)K\left(x,x_{i}\right)+b$$

### 2.2. Sparrow Search Algorithm

Whether SVM or SVR, the performance depended on the selection of the two parameters. In this paper, an SSA optimization algorithm is introduced to solve the hyperparameter problem and to improve the identification accuracy of mixed gas and the accuracy of gas-concentration prediction. SSA mainly simulates the foraging and anti-predatory behavior of sparrow groups. In the sparrow foraging process, sparrows are divided into finders and joiners according to their locations and energy reserves. The finders are responsible for finding food and for providing the joiners with foraging areas and directions. Joiners find food based on the information provided by the finders.

Assuming that there are sparrows, the sparrow population can be expressed as

(13) $$A=\left[\begin{array}{cccc} a_{1,1} & a_{1,2} & \ldots & a_{1,d}\\ a_{2,1} & a_{2,2} & \ldots & a_{2,d}\\ \vdots & \vdots & \vdots & \vdots \\ a_{p,1} & a_{p,2} & \ldots & a_{p,d} \end{array}\right]$$

where *p* is the number of sparrows, and *d* is the number of optimization parameters.

In the iterative process, the algorithm will continue to search for food within the search range if the finder does not find predators, and it will sound an alarm to alert other sparrows when the finder finds a predator. At this point, the whole flock of sparrows will quickly fly to other safe areas to feed. The location update of the discoverer is described as

(14) $$a_{i,j}^{t+1}=\left\{\begin{array}{c} a_{i,j}^{t}*\exp\frac{-i}{\beta *MaxT}\\ a_{i,j}^{t}+Q*L \end{array}\right.\begin{array}{c} if(R_{2}<ST)\\ if(R_{2}\geq ST) \end{array}$$

where *t* is the current number of iterations; MaxT is the maximum number of iterations; and $R^{2}$ and ST are the warning value and the safety value, respectively. When t<MaxT, SSA will sort the sparrows according to their fitness and find the current best and worst sparrows.

During foraging, the foraging strategy is determined by their energy level (fitness) of the sparrows. However, it will immediately leave the current location to obtain food if the joiner finds that the finder has found better food. At this time, the location update of the joiners is described as

(15) $$a_{i,j}^{t+1}=\left\{\begin{array}{c} Q*\exp\frac{a_{Worst}^{t}-a_{i,j}^{t}}{i^{2}}\\ a_{p}^{t+1}+\left|a_{i,j}^{t}-a_{p}^{t+1}\right|*\left(X^{T}{\left(XX^{T}\right)}^{-1}\right)*L \end{array}\right.\begin{array}{c} if(i<\frac{n}{2})\\ otherwise \end{array}$$

where $a_{p}$ is the best position currently occupied by the finder and $a_{Worst}$ is the current global worst position.

Sparrows with poor fitness and at the edge of the population are extremely vulnerable to natural enemies. These sparrows quickly fly to safety as soon as they are aware of the danger. However, once the sparrow in the middle of the population is aware of the danger, it will immediately move closer to other sparrows to reduce its own danger. For these sparrows, the location update is described as

(16) $$a_{i,j}^{t+1}=\left\{\begin{array}{c} a_{best}^{t}+\lambda \left|a_{i,j}^{t}-a_{best}^{t}\right|\\ a_{i,j}^{t}+K*\left(\frac{\left|a_{i,j}^{t}-a_{best}^{t}\right|}{\left(f_{i}-f_{w}\right)+\varepsilon }\right) \end{array}\right.\begin{array}{c} f_{i}>f_{g}\\ f_{i}=f_{g} \end{array}$$

where $a_{best}$ is the current global optimal position; λ is the step size; $f_{i}$ is the fitness of the current sparrow individual; and $f_{g}$ and $f_{w}$ are the current global best and worst fitness, respectively.

During the iteration process, if the sparrow’s new position is better than the previous position, the current position will be updated until the global optimal position and the global optimal fitness are found. During this period, the identity of the sparrow is also constantly updated and alternated. Every sparrow can be a finder if it is well adapted; however, the proportions of finders and joiners in the population are constant.

## 3. Experimental Results and Analysis

### 3.1. Data Preparation

This paper uses the data set “Gas sensor arrays in dynamic gas mixtures” from the UCI. The dataset contains 16 chemical sensors of four different types: TGS-2602, TGS-2602, TGS-2600, TGS-2600, TGS-2610, TGS-2610, TGS-2620, TGS-2620, TGS-2602, TGS-2602, TGS-2600, TGS-2600, TGS-2610, TGS-2610, TGS-2620, and TGS-2620. In the experiment, the sensor array composed of 16 gas sensors is placed in a 60 mL sealed box.

Gas samples are injected at a constant flow rate of 300 mL/min. The conductivity of the sensor (S/m) is continuously collected at a sampling frequency of 100 Hz. Each measurement is constructed by continuously acquiring signals from 16 sensor arrays. The concentration of the gas sample varies randomly. The gas samples are mainly composed of methane and ethylene in the air. The concentration range of ethylene is 0–20 ppm, and the concentration range of methane is 0–300 ppm. More details on the dataset can be found in [41]. After sorting and screening this dataset, 41,785 gas samples are finally selected for the experiment.

### 3.2. Mixed-Gas Identification

#### 3.2.1. Feature Extraction

The input data of this system is the output of 16 sensors in the sensor array. The output data is the gas-classification label, and the desired output is

(17) $$\left[\begin{array}{cccc} 1 & 0 & 0 & 0\\ 0 & 1 & 0 & 0\\ 0 & 0 & 1 & 0\\ 0 & 0 & 0 & 1 \end{array}\right]$$

which represents methane, ethylene, air, and mixed gases.

To improve the recognition accuracy of gas mixtures, the input data needs to be subjected to feature extraction. This eliminates or reduces the effect of the gas concentrations on the sensor array. As a common data analysis method, PCA can be used to extract the main feature vectors of data, achieve dimensionality reduction of high-dimensional data, and maximally preserve the feature information in high-dimensional data. Since the dimensions of the input data are inconsistent, which affects the calculation results, this study needs to be standardized to the original data first.

(18) $$a_{i}=\frac{x_{i}-\bar{x}}{\sqrt{\frac{1}{m}\sum_{i=1}^{m}{\left(x_{i}-\bar{x}\right)}^{2}}}$$

Standardization transforms the input data into data with a mean of 0 and a standard deviation of 1. This reduces the effect of outliers on the gas-classification results. Then, the input data is

(19) $$X=\left[\begin{array}{cccc} a_{1,1} & a_{2,1} & \ldots & a_{16,1}\\ a_{1,2} & a_{2,2} & \ldots & a_{16,2}\\ \vdots & \vdots & \vdots & \vdots \\ a_{1,41785} & a_{2,41785} & \ldots & a_{16,41785} \end{array}\right]=\left[\begin{array}{cccc} b_{1} & b_{2} & \ldots & b_{16} \end{array}\right]$$

where $b_{1}={\left[a_{1,1},a_{1,2},\ldots ,a_{1,41785}\right]}^{T}$.

After standardized processing, the variance matrix is represented as

(20) $$C=\frac{1}{41784}X^{T}X$$

The eigenvalue $k_{1},k_{2},\ldots ,k_{16}$ and $\lambda_{1},\lambda_{2},\ldots ,\lambda_{16}$ eigenvector of the covariance matrix are then obtained. The eigenvalues are sorted from large to small, and the contribution rate of each eigenvalue is

(21) $$p_{i}=\frac{\kappa_{i}}{\sum_{i=1}^{16}\kappa_{i}}$$

The cumulative contribution rate is obtained by solving the contribution rate of each eigenvalue. The cumulative contribution rate is the sum of the contribution rates of the first n eigenvalues. This is reflected the ability of the first *n* principal components to explain the original variable. Finally, the required principal components are obtained from the cumulative contribution rate: $X={\left(x_{1},x_{2},\ldots ,x_{n}\right)}^{T}$. The sixteen eigenvalues and contribution rates obtained from this data set after PCA processing are shown in Table 1.

As shown in Table 1, with the increase in the principal components, the increase in the contribution rate gradually decreases. When there are four principal components, the cumulative contribution rate reaches more than 99%. The four principal components explain more than 99% of the total variance. Thus, the original data can be reduced from 16 to 4 dimensions to represent the original features in this paper. After the data is reduced by PCA, the sample order of the classification dataset is randomly shuffled. According to the ratio of 8:2, this is divided into training sets and test sets.

#### 3.2.2. Classification Results for Mixed Gases

In the experiment, the value range of hyperparameter *c* and *g* of SVM are set as $\left[2^{-2}-2^{8}\right]$ and $\left[2^{-5}-2^{5}\right]$, respectively. The kernel function of SVM is the radial basis function. The related parameters of SSA are set as shown in Table 2.

In Table 2, *d* is the number of optimization parameters; *lb* is the lower limit of the optimization parameters in SVM; *ub* is the upper limit of the optimization parameters in SVM; *p* is the sparrow population size; *MaxT* is the maximum number of iterations; *ST* is the safety threshold; FD is the finder; JD is the joiner.

**Figure 3:**
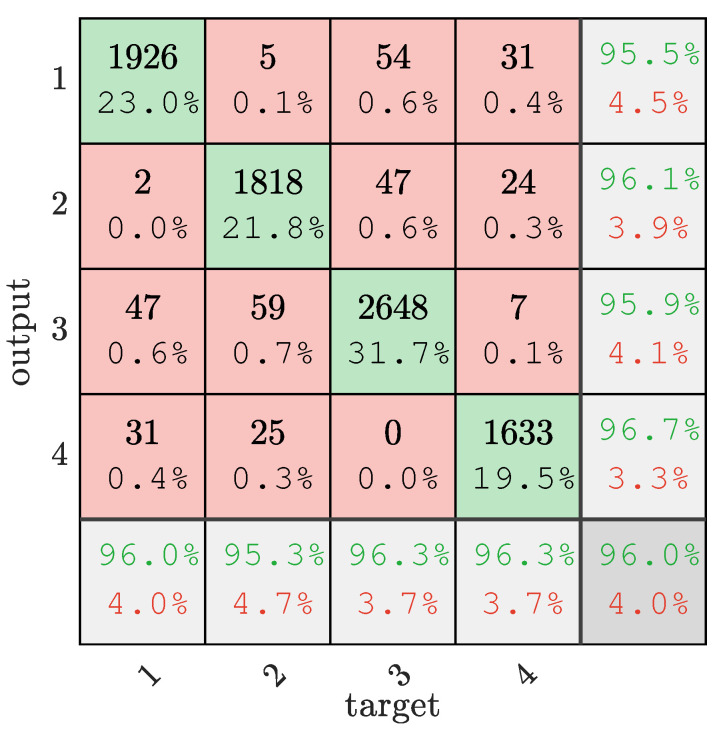
Mixed-gas-identification confusion matrix. ‘1’ is methane, ‘2’ is ethylene, ‘3’ is air, and ‘4’ is mixed gas.

After setting the relevant parameters of SSA, the optimal parameters *c* = 53.248 and *g* = 5.979 are obtained by solving Equations (14)–(16). Then, the best parameter combination is used to train on the training set to obtain the SSA-SVM model, and finally the classification test is performed on the test set. The classification results are shown in Figure 3.

It can be seen from Figure 3 that the accuracy rates of methane, ethylene, air, and mixed gas in the mixed-gas identification are 95.5%, 96.1%, 95.9%, and 96.7%, respectively. The accuracy rate is 96% among 2006 methane test samples, and 96.3% of samples were correctly identified as mixed gas among 1695 mixed-gas test samples. Overall, SSA-SVM correctly classified 8025 out of 8357 test samples. The accuracy rate reached 96%.

According to the model processing tasks, the evaluation criteria were also different. Accuracy is the most simple and intuitive evaluation index in classification problems. The accuracy is susceptible to the influence of the larger category if the sample is unevenly proportioned. In multi-classification, the arithmetic mean of the sample accuracy under each category is generally used as the evaluation index of the model.

(22) $$ACC=\sum_{i=1}^{4}\frac{TP_{i}}{TP_{i}+FP_{i}}$$

where TP is the number of correct predictions and FP is the number of incorrect predictions.

**Figure 4:**
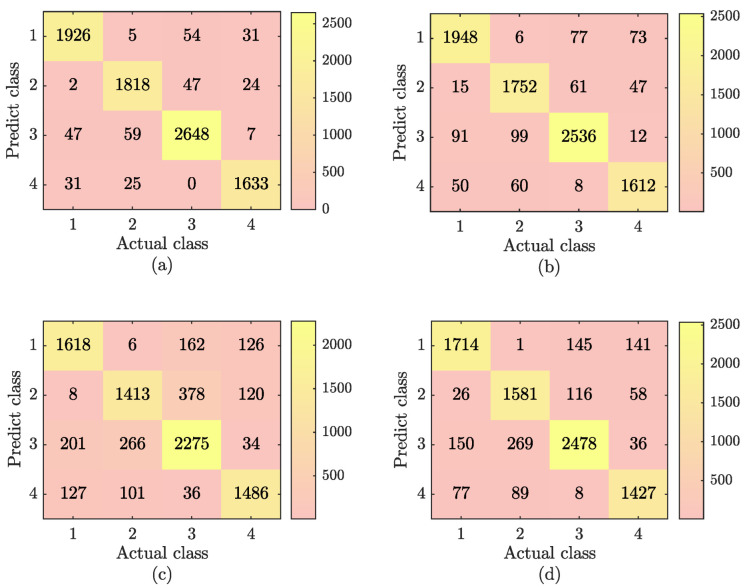
Mixed gas results for four mixed-gas-identification methods. (**a**) The SSA-SVM gas-identification results; (**b**) the RF gas-identification results; (**c**) the ELM gas-identification results; and (**d**) the BP neural network gas-identification results.

To further demonstrate the performance of SSA-SVM, we compared it with RF, ELM, and BP neural networks. The relevant parameters of the RF, ELM, and BP neural networks are shown in Table 3. The classification results are shown in Figure 4.

From the combination of Figure 4 and Equation (22), the accuracies of the four methods can be calculated as 96%, 92.9%, 86.8%, and 81.3%. SSA-SVM improved the accuracy by 3.1%, 8.2%, and 14.7% compared with the RF, ELM, and BP neural network.

**Figure 5:**
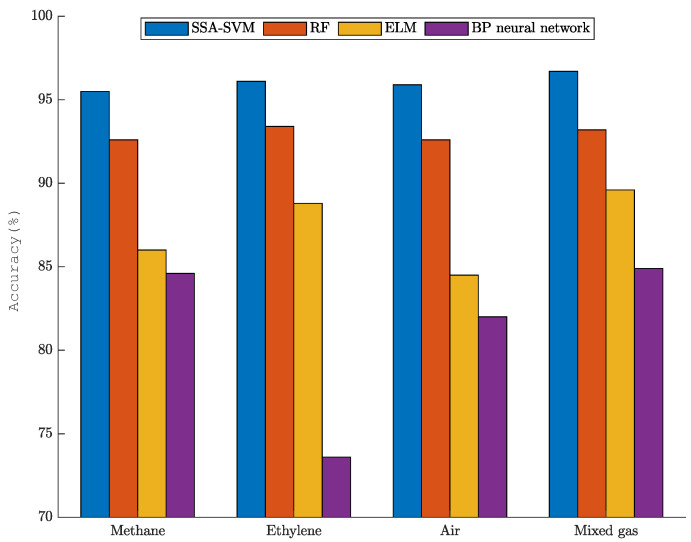
Gas identification accuracy of the four mixed-gas-identification methods.

In order to better reflect the mixed gas recognition ability of the SSA-SVM, RF, ELM, and BP neural networks, the accuracy rate of each category under the above methods is calculated. The comparison results are shown in Figure 5. As shown in Figure 5, the SSA-SVM method is better than the other three methods in identifying both single gases and mixed gases.

### 3.3. Prediction of Mixed-Gas Concentration

#### 3.3.1. Data Processing

In this paper, the characteristic subsets of each gas concentration are screened from the classification dataset. The input data is unchanged, and the output data is the gas concentration value. In the experiment, the input data and output data are normalized to be between 0 and 1 to improve the calculation efficiency of the regression model fitting process.

(23) $$y_{i}=\frac{x_{i}-\min_{1\leq j\leq n}\left\{x_{j}\right\}}{\max_{1\leq j\leq n}\left\{x_{j}\right\}-\min_{1\leq j\leq n}\left\{x_{j}\right\}}$$

Once the data process is completed, the sample order of the feature subset is randomly shuffled. It is divided into a training set and test set with a ratio of 9:1, and five-fold cross-validation will be performed during the training process. The sample sizes for each subset of gas concentration characteristics are shown in Table 4.

#### 3.3.2. Mixed-Gas-Concentration Prediction Results

The value range of the hyperparameter *q* is $\left[2^{-5}-2^{10}\right]$, and the value range of *t* is $\left[2^{-5}-2^{10}\right]$. The loss function value is set as 0.01. The relevant parameters of SSA are set as Table 5.

In Table 5, *d* is the number of optimization parameters; *lb* is the lower limit of optimization parameters in SVR; *ub* is the upper limit of optimization parameters in SVR; *p* is the sparrow population size; *MaxT* is the maximum number of iterations; *ST* is the safety threshold; FD is the finder; JD is the joiner.

According to Equations (14)–(16), the two hyperparameters of SVR are optimized, and the optimal parameter combination is finally determined. The optimal parameter combination of each feature subset is shown in Table 6.

**Figure 6:**
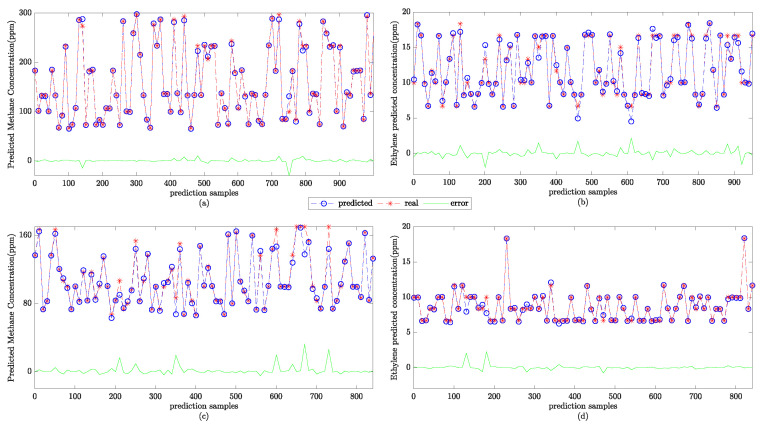
The SSA-SVR gas-concentration prediction results. (**a**) The predicted concentration of methane under a single gas; (**b**) the predicted concentration of ethylene under a single gas; (**c**) the predicted concentration of methane under the mixed gas; and (**d**) the predicted concentration of ethylene in the mixed gas.

Then, the gas-concentration-prediction test is conducted using the test set. The prediction results are normalized to obtain the final concentration prediction results. The SSA-SVR gas-concentration-prediction results are shown in Figure 6.

The coefficient of determination $R^{2}$ is one of the performance evaluation indicators of the regression model, which is applied to reflect the accuracy of the regression model to fit the data. The value range of $R^{2}$ is 0–1. The closer the value is to 1, the better the model fits the data.

(24) $$R^{2}=1-\frac{\sum_{i=1}^{n}{\left(y_{i}-f\left(i\right)\right)}^{2}}{\sum_{i=1}^{n}{\left(y_{i}-\bar{y}\right)}^{2}}*100\%$$

**Figure 7:**
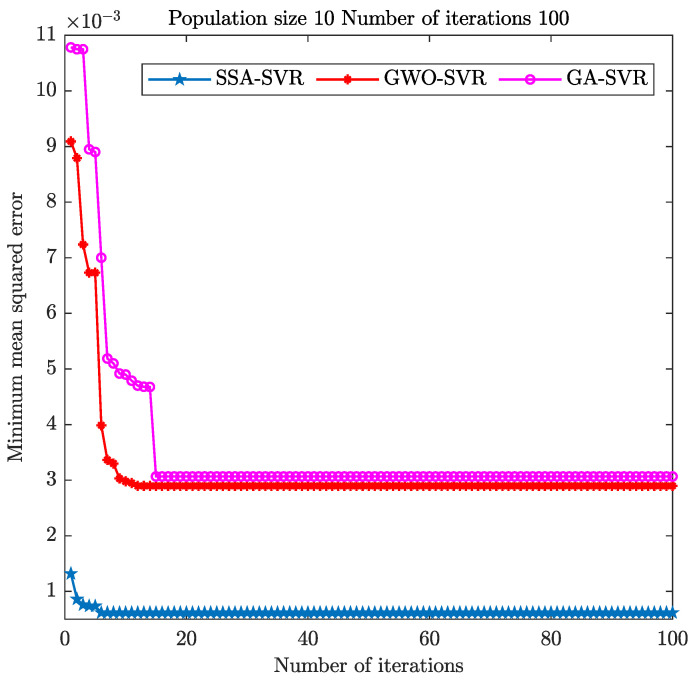
The mean square error of the three optimization algorithms.

To further validate the regression effect of the SSA algorithm for optimizing SVR, we compared it with the GWO and GA optimization algorithms. The minimum mean square error result is shown in Figure 7. The relevant parameter settings of the GWO and GA optimization algorithms are shown in Table 7 and Table 8.

In Table 7, *SA* is the number of wolves, *Maxt* is the maximum number of iterations, *dim* is the number of optimization parameters, *lb* is a lower bound on the optimization parameters, and *ub* is an upper bound on the optimization parameters.

In Table 8, *Nind* is the population size; *Maxgen* is the maximum number of genetic generations; *ggap* is the generation gap; *px* and *pm* are the crossover probability and variation probability, respectively; and *select* is the selection function.

The mean square error of the SSA optimization algorithm reaches the minimum after six iterations under the same population size and iteration numbers. The GWO optimization algorithm achieves the minimum mean square error in about 12 iterations, and the GA optimization algorithm takes about 15 iterations. In comparison, the convergence speed of the SSA optimization algorithm is better than that of the GWO and GA algorithms.

**Figure 8:**
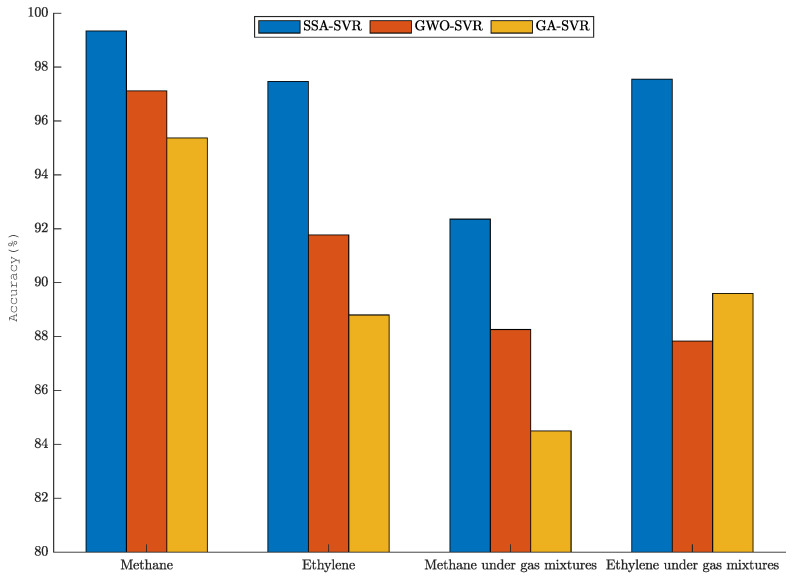
Gas-concentration-prediction fits.

In order to further intuitively reflect the fitting degree of the prediction data of SSA-SVR, GWO-SVR, and GA-SVR, the determination coefficient of each model is calculated. The fitting results of the gas-concentration prediction are shown in Figure 8. Whether for a single gas or a mixture of gases, the concentration prediction fit of SSA-SVR is better than that of GWO-SVR and GA-SVR. The fitting degree of SSA-SVR for single gas-concentration prediction is over 97%. The fitting degrees of SSA-SVR for the prediction of methane and ethylene concentrations under mixed gas are 92.36% and 97.55%.

## 4. Conclusions

Mixed-gas detection is of great importance in industrial production processes. This type of detection is important to ensure the green development and safe production of enterprises. This paper presents a mixed-gas-identification method based on SSA-SVM. Our conclusions are summarized as follows.

(1) In the mixed-gas identification experiment, SSA-SVM was combined with PCA for mixed-gas identification. PCA was adopted to reduce the original data from 16 dimensions to 4 dimensions to achieve dimensionality reduction of the high-dimensional data. The influence of redundant data on the SSA-SVM model was reduced. The classification accuracy of SSA-SVM on the test set was over 96%. Compared with the RF, ELM, and BP neural network models, the classification accuracy of SSA-SVM was improved by 3.1%, 8.2%, and 14.7%, respectively.

(2) In the gas-concentration-prediction experiment, the prediction fit and convergence iteration speed of SSA-SVR were better than those of GWO-SVR and GA-SVR. For the concentration prediction of a single gas, the fitting degree of SSA-SVR was more than 97%. The fitting degree of the predicted concentration of methane was as high as 99.34%. The fitting degrees of the predicted concentrations of methane and ethylene in mixed gas reached 92.36% and 97.55%.

(3) With the long-term use of the sensor, there will inevitably be sensor drift. Data distortion is prone to affect data analysis. Next, we will focus on this issue and reduce the effect of drift on the system.

## Figures and Tables

**Table 1:** Eigenvalues and the contribution rates of 16 eigenvectors.

Principal Components	Eigenvalue	Contribution Rate%	Cumulative Contribution Rate%
PC1	10.650	66.562	66.562
PC2	3.779	23.620	90.182
PC3	1.067	6.669	96.851
PC4	0.413	2.585	99.436
PC5	0.039	0.246	99.682
PC6	0.036	0.224	99.906
PC7	0.007	0.049	99.955
PC8	0.003	0.017	99.972
PC9	0.002	0.012	99.984
PC10	0.001	0.006	99.990
PC11	0	0.006	99.996
PC12	0	0.002	99.998
PC13	0	0.001	99.999
PC14	0	0.001	1
PC15	0	0	1
PC16	0	0	1

**Table 2:** The SSA-related parameters in SSA-SVM.

Parameter	*d*	*lb*	*ub*	*p*	*Max*T	*ST*	*FD*	*JD*
Value	2	$\left[2^{-2},2^{-5}\right]$	$\left[2^{8},2^{5}\right]$	50	100	0.6	90%	10%

**Table 3:** Related parameters of the comparative test.

Method	Related Parameters
RF	trees = 500, mtry = 4
ELM	the number of hidden layer neurons is 100
BP-NN	the number of neurons in the hidden layer is 10 the activation function is Sigmoid, three layers

**Table 4:** The number of feature subset samples.

Data	Single Gas		Mixed Gas	
	CH4	C2H4	CH4	C2H4
Train	9010	8589	7637	7637
Test	1002	955	849	849

**Table 5:** SSA-related parameters in SSA-SVR.

Parameter	*d*	*lb*	*ub*	*p*	*MaxT*	*ST*	*FD*	*JD*
Value	2	$\left[2^{-5},2^{-5}\right]$	$\left[2^{10},2^{10}\right]$	50	100	0.8	40%	60%

**Table 6:** The best parameter combination.

Parameter	Single Gas		Mixed Gas	
	CH4	C2H4	CH4	C2H4
*q*	66.6915	5.9072	14.8780	11.9311
*t*	7.8229	66.3515	13.8491	32.6786

**Table 7:** The parameters related to the GWO optimization algorithm.

Parameter	*SA*	*Maxt*	*dim*	*lb*	*ub*
Value	50	100	2	$\left[2^{-5},2^{-5}\right]$	$\left[2^{10},2^{10}\right]$

**Table 8:** The parameters related to the GA optimization algorithm.

Parameter	*Nind*	*Maxgen*	*ggap*	*px*	*pm*	*select*
Value	50	100	0.9	0.7	0.1	rws

## Data Availability

Data are available in a publicly accessible repository that does not issue DOIs. Publicly available datasets were analyzed in this study. These data can be found here: https://archive-beta.ics.uci.edu, (accessed on 30 September 2022).
